# Magnitude of troponin elevation in patients with biomarker evidence of myocardial injury: relative frequency and outcomes in a cohort study across a large healthcare system

**DOI:** 10.1186/s12872-023-03168-0

**Published:** 2023-03-24

**Authors:** Colleen K. McIlvennan, Manuel Urra, Laura Helmkamp, John C. Messenger, David Raymer, Karen S. Ream, J. Bradley Oldemeyer, Amrut V. Ambardekar, Kathleen Barnes, Larry A. Allen

**Affiliations:** 1 Department of Medicine, School of Medicine, University of Colorado Anschutz Medical Campus, 12631 East 17 Avenue, B130, Aurora, CO 80045 USA; 2 Adult and Child Center for Outcomes Research and Delivery Science (ACCORDS), School of Medicine, University of Colorado Anschutz Medical Campus, Aurora, CO USA; 3 University of Colorado Health, Fort Collins, CO USA; 4 Colorado Center for Personalized Medicine, School of Medicine, University of Colorado Anschutz Medical Campus, Aurora, CO USA

**Keywords:** Troponin, Biomarkers, Myocardial infarction, Risk, Heart failure

## Abstract

**Background:**

Serum troponin levels correlate with the extent of myocyte necrosis in acute myocardial infarction (AMI) and predict adverse outcomes. However, thresholds of cardiac troponin elevation that could portend to poor outcomes have not been established.

**Methods:**

In this cohort study, we characterized all cardiac troponin elevations > 0.04 ng/mL (upper limit of normal [ULN]) from patients hospitalized with an ICD-9/10 diagnosis of AMI across our health system from 2012–2019. We grouped events into exponential categories of peak cardiac troponin and evaluated the association of these troponin categories with all-cause mortality, heart transplants, or durable left ventricular assist devices (LVAD). Patients with cardiac troponin > 10,000 × ULN were manually chart reviewed and described.

**Results:**

There were 18,194 AMI hospitalizations with elevated cardiac troponin. Peak troponin was 1–10 × ULN in 21.1%, 10–100 × ULN in 34.8%, 100–1,000 × ULN in 30.1%, 1,000–10,000 × ULN in 13.1%, and > 10,000 × ULN in 0.9% of patients. One-year mortality was 17–21% across groups, except in > 10,000 × ULN group where it was 33% (adjusted hazard ratio (99%CI) for > 10,000 × ULN group compared to all others: 1.86 (1.21, 2.86)). Hazards of one-year transplant and MCS were also significantly elevated in the > 10,000 × ULN group.

**Conclusions:**

Elevation in cardiac troponin levels post AMI that are > 10,000 × ULN was rare but identified patients at particularly high risk of adverse events. These patients may benefit from clarification of goals of care and early referral for advanced heart failure therapies. These data have implications for conversion to newer high-sensitivity cardiac troponin assays whose maximum assay limit is often lower than traditional assays.

**Supplementary Information:**

The online version contains supplementary material available at 10.1186/s12872-023-03168-0.

## Introduction

Cardiac specific troponins, including Troponin T and Troponin I, are regulatory proteins unique to the myocardium. The serologic concentration of these proteins rise and fall in the setting of myocardial injury [1]. The utility of cardiac troponin for the diagnosis of acute myocardial infarction (AMI) and other forms of myocardial injury is well established and now incorporated into the Fourth Universal Definition of Myocardial Infarction [2].

Several studies have shown a correlation between the magnitude of cardiac troponin elevation and the extent of myocyte necrosis, the development of heart failure, and the risk of mortality [3–7]. However, despite the ubiquitous use of troponins in healthcare for more than a decade, detailed analyses of troponin levels and clinical outcomes are surprisingly sparse. As such, thresholds of troponin elevation that could trigger particular clinical action have not been thoroughly explored or well developed.

Patients with massive myocardial injury are at high risk for adverse outcomes, including death. However, the clinical course can be variable and difficult to predict. These patients may be eligible for durable left ventricular assist device (LVAD) or cardiac transplantation, but getting patients to such therapies usually requires timely engagement of an advanced heart failure program, sometimes with early initiation of temporary circulatory support. Late referral after massive myocardial infarction when patients have progressed to multi-organ failure can cause patients to “miss the window” for advanced therapies [8].

Given the known relationship of cardiac troponin levels to degree of cardiomyocyte injury and based on anecdotal cases of patients whose cardiac troponin level was greater than assay maximum, we hypothesized that markedly elevated peak cardiac troponin is associated with particularly high rates of adverse outcomes. Better characterizing this relationship of very high cardiac troponin levels to outcomes could improve rapid triage of in-hospital care, guide post-discharge planning, and encourage completion of advance care directives for this high-risk population. In addition, if an absolute threshold of cardiac troponin elevation could be identified beyond which outcomes were worse than that seen with LVAD and transplant, a simple rule of thumb could be created to prompt immediate consideration for engagement of advanced heart failure or palliative care consultation.

Therefore, we aimed to systematically characterize all cardiac troponin elevations across a diverse health system and compare absolute cardiac troponin levels to 1-year outcomes (death, mechanical circulatory support (MCS), and heart transplant) in these populations.

## Methods

### Study population and data sources

UCHealth includes 12 hospitals and over 30 clinics within Colorado that cover 19% of the 5.8 million Coloradans, as well as referrals from relatively rural areas in surrounding states. Major efforts were made in 2011 to convert the entire system to a single instance of the Epic electronic health record for all inpatient and outpatient activities. Since 2011, all laboratory test results, as well as diagnoses, vital signs, procedures, encounters, and deaths are reported in the UCHealth Epic record and then downloaded into the Health Data Compass virtual data warehouse (https://www.healthdatacompass.org/). All procedures were approved by the Colorado Institutional Review Board and were conducted in accordance with the principles outlined in the Declaration of Helsinki.

Health Data Compass was queried for all hospital admissions to the UCHealth between 2012–2019 for patients with a diagnosis of acute myocardial infarction (AMI) based on International Classification of Disease, Ninth Revision, Clinical Modification (ICD-9) (410, 414) and ICD-10 (I-21, I-22, I-25) codes. Based on this cohort, additional data collected were demographics (age, gender, race/ethnicity), labs obtained during AMI encounters (highest troponin, highest serum creatinine), and primary outcomes in the following year after the hospitalization event (death, MCS, heart transplant by Current Procedural Terminology codes).

All hospitals used a standardized “Chest Pain Protocol” pathway, which recommended cardiac troponin testing at presentation, 3 h, and 6 h. During the study period, the cardiac troponin assay most commonly used for clinical diagnosis across hospitals was the ADIVA Centaur cardiac troponin I assay. The reference range for the assay was 0.0–0.04 ng/mL. A minority of troponin values reflected the use of alternative assays, primarily the Ortho Clinical Diagnostics VITROS 5600 (reference range 0.00–0.03 ng/mL). Upper limit of normal (ULN) was determined as above the 99^th^ percentile of the target population. However, troponin data stored in the electronic health record did not include assay details. Within this context, we used a singular cutoff of 0.04 ng/mL for this analysis. This approach ensured patients met criteria for the Fourth Universal definition of myocardial injury (i.e. a serum cardiac troponin test result > 99^th^ percentile of the ULN). This approximation also reflected chest pain-myocardial infarction treatment pathways at all health system sites which used a cutoff of 0.04 ng/mL regardless of assay. We limited our patient selection to age ≥ 18 years. For patients with multiple events, all events and associated cardiac troponin were considered.

For the subgroup of patients with cardiac troponin > 10,000 times the ULN (> 400 ng/dL), the clinical course was further explored via manual chart review (by MU, CKM, LAA). Data obtained included MI type, procedures, and types of temporary MCS utilized, as well as confirmation of death, durable MCS, and heart transplant.

### Patient and public involvement

Patients and/or the public were not involved in the design, conduct, reporting, or dissemination plans of this research.

### Statistical analysis

Patients were described using counts and percentages, both overall and by cardiac troponin level. cardiac troponin was grouped into exponential categories: 1–10 × ULN (0.04–0.4 ng/mL], 10–100 × ULN (0.4–4 ng/mL], 100–1,000 × ULN (4–40 ng/mL], 1,000–1,0000 × ULN (40–400 ng/mL], and > 10,000 × ULN (> 400 ng/mL). Initial survival comparisons between groups were performed using Kaplan–Meier plots, and Kaplan–Meier estimates were used to obtain outcome rates at 1 year; death estimates account for censoring at the time of data collection for patients with recent events, while other estimates account for censoring both at the time of data collection and due to death. Hazard Ratios (HRs) and associated confidence intervals (CIs) for each troponin category compared to the reference category were obtained via Cox models, with robust sandwich covariance matrix estimates to account for the correlation among multiple events for some subjects. Analyses were repeated with the addition of several potential confounders–age, race, ethnicity, diabetes, hypertension, smoking status, and creatinine level– added to the model to obtain adjusted hazard ratios. Finally, all analyses were repeated with lowest 4 categories combined, compared with the > 10,000 × ULN (> 400 ng/mL) group, to provide hazard for this group compared to all other subjects. CIs presented are 99% confidence intervals, corresponding to an alpha of 0.01. Analyses were conducted in SAS version 9.4.

## Results

### Patient characteristics

Between 2012 and 2019, 27,570 hospitalizations occurred where a primary diagnosis of AMI was assigned. Of these, 8,210 (30%) did not have a cardiac troponin value recorded, and 1,166 (4%) had a peak cardiac troponin value ≤ 0.04, resulting in an analytic sample of 18,194 events. Some patients had multiple events in the dataset, resulting in a patient-level sample of 15,800 unique patients (Supplement). Baseline characteristics of patients at each event are reported in Table 1. Most patients were male, Caucasian, and older, with almost a third actively smoking cigarettes.

**Table 1:** Patient characteristics by troponin category at the event level (patients with more than one myocardial infarction can be included more than once)

	**All**	**TnI (0.04–0.4] *n *= 3,833** **% (N)**	**TnI (0.4–4] *n* = 6,326** **% (N)**	**TnI (4–40] *n* = 5,485** **% (N)**	**TnI (40–400] *n* = 2,382** **% (N)**	**TnI 400 + *n* = 168** **% (N)**
Female gender	38.6% (7,020)	42.4% (1,625)	45.1% (2,853)	34.6% (1,899)	25.6% (609)	20.2% (34)
Race						
White or Caucasian	75.9% (13,818)	73.3% (2,809)	77.3% (4,889)	77.0% (4,224)	74.5% (1,774)	72.6% (122)
Black or African American	8.6% (1,571)	11.7% (449)	8.8% (558)	7.3% (402)	6.3% (151)	6.5% (11)
Other or multiple races	13.1% (2,379)	13.2% (505)	12.0% (757)	12.9% (710)	15.9% (379)	16.7% (28)
Unknown	2.3% (426)	1.8% (70)	1.9% (122)	2.7% (149)	3.3% (78)	4.2% (7)
Ethnicity						
Non-Hispanic	87.0% (15,833)	87.2% (3,344)	88.3% (5,587)	86.3% (4,732)	85.5% (2,037)	79.2% (133)
Hispanic	10.5% (1,911)	10.7% (409)	9.7% (615)	10.8% (593)	11.3% (268)	15.5% (26)
Unknown	2.5% (450)	2.1% (80)	2.0% (124)	2.9% (160)	3.2% (77)	5.4% (9)
Age						
< 50	12.5% (2,282)	11.7% (448)	11.1% (701)	13.6% (748)	15.1% (359)	15.5% (26)
50–64	32.3% (5,871)	30.2% (1,156)	29.8% (1,883)	33.8% (1,853)	38.1% (908)	42.3% (71)
65–79	36.9% (6,713)	36.0% (1,381)	38.6% (2,441)	36.7% (2,013)	34.4% (819)	35.1% (59)
80 +	18.3% (3,328)	22.1% (848)	20.6% (1,301)	15.9% (871)	12.4% (296)	7.1% (12)
Diabetes	1.9% (345)	1.7% (66)	2.2% (140)	1.8% (99)	1.6% (37)	1.8% (3)
Hypertension	3.8% (700)	3.8% (146)	4.1% (260)	4.0% (222)	2.9% (68)	2.4% (4)
Cigarettes	29.2% (5,312)	32.3% (1,238)	30.3% (1,918)	26.8% (1,471)	26.8% (639)	27.4% (46)
Highest creatinine > 1.5	29.7% (5,400)	30.6% (1,173)	30.3% (1,914)	29.6% (1,624)	25.5% (608)	48.2% (81)

### Distribution of Peak troponin values

**Fig. 1:**
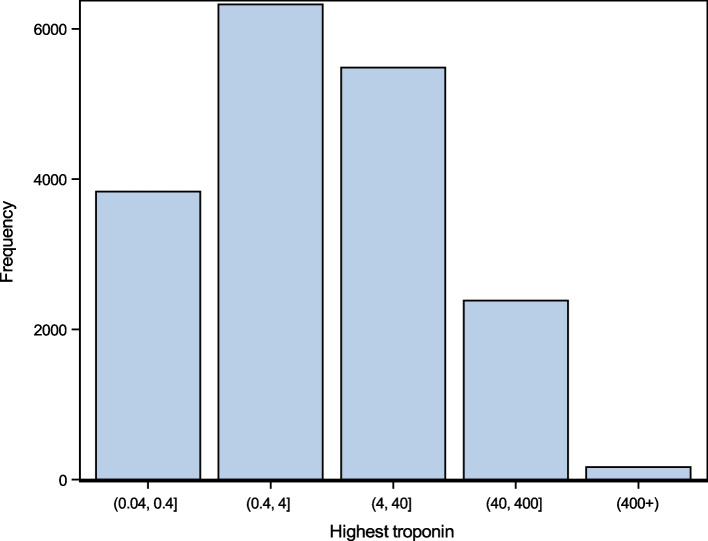
Histogram of exponential groupings of peak serum troponin elevations (ng/dL) among hospitalizations for acute myocardial infarction 2012–2019

Of the 18,194 events with a diagnosis of AMI and a recorded cardiac troponin above ULN, 21.1% (*n* = 3,833) had a peak cardiac troponin 1–10 × ULN (0.04–0.4 ng/mL], 34.8% (*n* = 6,326) had a peak cardiac troponin 10–100 × ULN (0.4–4 ng/mL], 30.1% (*n* = 5,485) had a peak cardiac troponin 100–1,000 × ULN (4–40 ng/mL], 13.1% (*n* = 2,382) had peak a cardiac troponin 1,000–10,000 × ULN (40–400 ng/mL], and 0.9% (*n* = 168) had peak troponin > 10,000 × ULN (> 400 ng/mL), (Fig. 1).

### Treatments and outcomes by Peak troponin

**Fig. 2:**
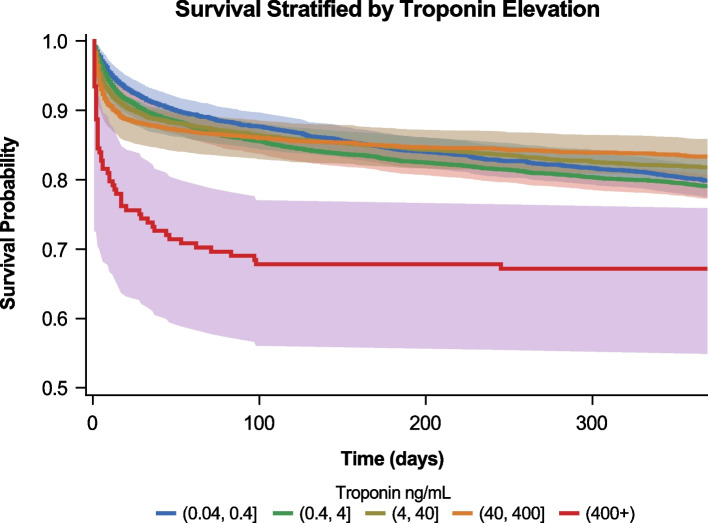
One-year survival in patients with hospitalization for acute myocardial infarction, stratified by exponential groupings of peak serum troponin elevation

At 1 year, mortality rates were highest with highest levels of cardiac troponin (Fig. 2). Of the patients with a cardiac troponin > 10,000 ULN, 33% died within one year (Table 2). The other groups had mortality rates ranging from 17–21%; adjusted hazard ratio (AHR) (99% CI) for the cardiac troponin > 10,000 ULN group compared to all others was 1.86 (1.21, 2.86). MCS in the year after the event increased with cardiac troponin level, highest in patients with cardiac troponin > 10,000 × ULN when reviewed using automated data extraction; the AHR (99% CI) for the cardiac troponin > 10,000 ULN group compared to all others was 5.73 (3.38, 9.70). In patients with a cardiac troponin > 10,000 × ULN, 1.6% (*n* = 2) received durable LVAD placement and 2.5% (*n* = 3) received a heart transplant within one year. Fewer than 1% of patients in the other groups were noted to have received a LVAD or heart transplant within one year. For LVAD this difference was not statistically significant; for transplant the AHR (99% CI) for the cardiac troponin > 10,000 ULN group compared to all others was 17.04 (3.00, 96.86).

**Table 2:** One-year outcomes comparison between troponin groups

**Outcome**		**[0.04,0.4]**	**(0.4,4]**	**(4,40]**	**(40,400]**	**(400 +)**	**HR (99% CI)** **For 400 + compared to other groups combined**
Death	% at 1 year	20.0%	20.9%	18.2%	16.7%	32.8%	
	HR (99% CI)	1.06 (0.93, 1.21)	1.14 (1.02, 1.27)	-reference-	0.93 (0.80, 1.09)	2.13 (1.46, 3.13)	2.03(1.40, 2.96)
	Adj. HR (99%CI)	0.95 (0.84, 1.08)	1.04 (0.93, 1.16)	-reference-	1.08 (0.92, 1.26)	1.89 (1.22, 2.92)	1.86 (1.21, 2.86)
Left ventricular assist device	% at 1 year	0.3%	0.2%	0.4%	0.4%	1.6%	
	HR (99% CI)	0.84 (0.26, 2.72)	0.55 (0.22, 1.39)	-reference-	0.97 (0.34, 2.75)	4.03 (0.59, 27.50)	5.01 (0.78, 32.25)
	Adj. HR (99%CI)	0.75 (0.26, 2.22)	0.54 (0.22, 1.31)	-reference-	1.04 (0.36, 3.01)	3.31 (0.46, 23.80)	4.16 (0.62, 27.80)
Transplant	% at 1 year	0.0%	0.0%	0.3%	0.2%	2.5%	
	HR (99% CI)	0.00 (0.00, 0.00)	0.07 (0.01, 0.82)	-reference-	0.70 (0.19, 2.64)	8.92 (1.66, 47.86)	20.96 (3.97, 110.64)
	Adj. HR (99%CI)	0.00 (0.00, 0.00)	0.07 (0.01, 0.71)	-reference-	0.68 (0.16, 2.81)	7.62 (1.32, 44.17)	17.04 (3.00, 96.86)
Mechanical circulatory support	% at 1 year	1.2%	1.6%	3.0%	4.5%	16.2%	
	HR (99% CI)	0.4 (0.26, 0.63)	0.53 (0.38, 0.75)	-reference-	1.55 (1.12, 2.15)	5.78 (3.30, 10.16)	7.42 (4.35, 12.68)
	Adj. HR (99%CI)	0.43 (0.27, 0.67)	0.55 (0.39, 0.77)	-reference-	1.59 (1.14, 2.21)	4.70 (2.71, 8.16)	5.73 (3.38, 9.70)

Survival estimates obtained via Kaplan-Meier. Hazard ratios obtained via Cox models, with robust sandwich covariance matrix estimates to account for the correlation among multiple events for some subjects. Covariates in adjusted models included age, gender, race, ethnicity, diabetes, hypertension, smoking status, and creatinine level.

### Highest troponin group

For all 168 patients with cardiac troponin > 10,000 ULN (> 400 ng/dL), the clinical course was further explored via chart review (Table 3). Almost all patients were diagnosed with a Type 1 MI (95%, *n* = 158). Most patients received a PCI within 7 days of cardiac troponin peak (87%, *n* = 146) and only 5 patients received CABG within 7 days (3%). In our manual chart review performed in this group, it was noted that 42 MCS procedures performed prior to transfer between hospitals were not captured by the automatic data extraction leading to a difference between patients requiring temporary MCS in the data extraction at 16% vs 41% when manual chart review was performed in this group. For those *n* = 69 (41%) who received temporary MCS during the hospitalization, *n* = 40 (24%) received intra-arterial balloon placement, *n* = 27(16%) received Impella support, and *n* = 10 (6%) needed extracorporeal membrane oxygenation. Five deaths were picked up in the > 400 ng/mL group through manual chart review which were missed during automated data extraction.

**Table 3:** Outcomes obtained via chart review for 168 pts in highest troponin group only

	**% (N) of 168 patients**
**Type of myocardial infarction**	
Type 1	94% (158)
Type 2	5% (8)
Unknown	1% (2)
**Procedure (within 7 days)**	
Percutaneous coronary intervention within 7 days	87% (146)
Coronary artery bypass graft within 7 days	3% (5)
Troponin peak occurred post-procedure	79% (134)
**Outcomes (within 1 year)**	**% w/ outcome (KM estimate at 1 year)**
Death	36%
Any temporary Mechanical circulatory support	41%
Intra-aortic balloon pump	24%
Impella	16%
Extracorporeal membrane oxygenation	6%
Tandem Heart	0%
Other	2%

MCS types can add up to more than ‘any MCS’ because a patient can have more than one type of temporary MCS

Procedures information is simple *N* and percent among this group. Outcomes information obtained via Kaplan–Meier estimates to account for censoring

## Discussion

This retrospective observational study aimed to identify whether a threshold peak cardiac troponin level at the time of AMI could quickly identify patients at particularly high risk of subsequent adverse outcomes, and thus rapid consideration of MCS, LVAD placement, heart transplant, or involvement of palliative care. We identified that an inflection point indicating much worse outcomes exist in patients with cardiac troponin levels elevated > 10,000 times the ULN (> 400 ng/mL). Though this is an infrequent occurrence, accounting for only 1% of all our cases, this group had a statistically higher incidence of death at 33% vs 17–21% in all other groups (AHR (99% CI): 1.86(1.21, 2.86)) and captured all use of advanced therapies (transplant and LVAD) in the following year.

Although there is a well understood relationship between the magnitude of troponin elevation and the extent of myocyte necrosis [9], the quantifiable risk of mortality and requirement of advanced therapies has not been previously explored. This study is the first to our knowledge to stratify the need for MCS, LVAD or transplant based on extent of troponin elevation during AMI. Only one other study of post PCI patients correlated very high troponin levels with death [7]. Due to the worsened outcomes in patients with a cardiac troponin > 10,000 × ULN (> 400 ng/mL), this frequently used biomarker of myocardial necrosis may serve to identify patients that would benefit from early consideration of advanced cardiac therapies. There are several triggers for patients to be referred for consideration of advanced therapies, including the I-NEED-HELP acronym [10]. Our analysis serves as possibly another “trigger” for consideration to refer patients for advanced therapies, in the context of individual preference and consideration of comorbid conditions. In the subset of our study population having a troponin > 400 ng/ dL (*n* = 168), one third died, more than double the rate of death after transplant or durable LVAD implantation. We therefore conclude that early consideration and referral for advanced therapies in this small sub-set of patients would be reasonable.

Peak troponin was noted in the post-PCI period for most patients in the highest troponin group (79%, *n* = 132). This group was noted to have significantly worse outcomes, including mortality, than the more modest troponin elevations. This is a noteworthy observation as there exists uncertainty as to the clinical significance of periprocedural cardiac biomarker elevation [11], with many providers considering the degree of biomarker elevation after PCI to have minimal prognostic significance. We recognize that some providers do not check troponin levels after PCI, such that the patients included here may represent a higher risk population (e.g. ongoing shock, complex PCI). Regardless, the data collected in our study, as well as another recent study [7], suggest post-procedural troponin elevation may be a clinically relevant datapoint.

Troponin assays and their relationship to novel therapies for revascularization, inflammatory response, and infarct size is evolving [12]. Use of high sensitivity troponin assays (HsTrop) are being widely adopted given superior sensitivity to traditional troponin assays for early detection of acute coronary syndrome [13, 14]. Though there is improved detection of low levels of troponin with HsTrop assays, the upper range of contemporary HsTrop assays is significantly lower than traditional troponin assays (e.g., 25,000 ng/L for Siemens HsTrop and 270,270 ng/L for Beckman HsTrop, compared to 500,000 ng/L for the traditional Beckman cardiac troponin assay used for many of the patients in this study). This loss of quantitation at the upper ranges of troponin release with HsTrop may have implications for prognosis after AMI, and future iterations of HsTrop assays may need to consider optimizing both low and high ends of troponin quantitation. In the meantime, similar studies to this one with HsTrop results may be instructive.

### Limitations

First, as with all retrospective studies, we were limited by the quality of available data for analysis. We used ICD codes to identify patients with AMI and may have missed patients that had cardiac troponin elevations that did not meet criteria for AMI coding. Many true peak cardiac troponins are missed as post-PCI measurement of troponins is variable. Second, automated data pulls of the electronic health record also have varying degrees of inaccuracy (e.g., 5 deaths were picked up in the > 400 ng/mL group through manual chart review which were missed during automated data extraction). Third, low event numbers for some outcomes, particularly LVAD and transplant, resulted in wide confidence intervals and limited precision in our estimates and between-group comparisons. Fourth, our estimation of hazards of MCS, transplant, and LVAD in the presence of the competing risk of death assumes that events are independent, and our hazard ratios may be altered to the extent that this assumption is not met. Fifth, we only assessed cardiac troponin I and not other cardiac troponins. The goal of our study was not to assess the relative performance of multiple assays, but rather to assess the prognostic information conferred by orders of magnitude serum troponin elevation above ULN. Last, HsTrop are currently being increasingly adopted and traditional troponin assays will likely be phased out in the future.

## Conclusion

Overall, cardiac troponin > 10,000 times the ULN (> 400 ng/mL), though rare, has particularly poor outcomes in regard to requiring mechanical circulatory support and death. This serves to identify a group where clarification of goals of care and consideration for early referral for advanced therapies may prove valuable. Here we also highlight the relevance of measuring cardiac troponin until peak even if patients undergo PCI. Current HsTrop assays that are being increasingly adopted, often have lower upper limits of quantification, which may lead to a loss of prognostic information after AMI.

## Supplementary Information

**Additional file 1: Supplemental Table 1.** Patient-level and event level demographics.

## Data Availability

The datasets used and/or analyzed during the current study are available from the corresponding author on reasonable request.

## Abbreviations

AMI: Acute myocardial infarction
CABG: Coronary artery bypass graft
HsTrop: High sensitivity troponin
ICD-9,10: International Classification of Disease
LVAD: Left ventricular assist device
MCS: Mechanical circulatory support
PCI: Percutaneous coronary intervention
ULN: Upper limit of normal
