# Supplementary material for: Magnitude of troponin elevation in patients with biomarker evidence of myocardial injury: relative frequency and outcomes in a cohort study across a large healthcare system
Source: BMC Cardiovasc Disord. 2023 Mar 24;23:151. doi: 10.1186/s12872-023-03168-0 (PMC10037877; doi:10.1186/s12872-023-03168-0)
Supplement: Supplementary file 1 — Additional file 1: Supplemental Table 1. Patient-level and event level demographics. [file 12872_2023_3168_MOESM1_ESM.docx]

# SUPPLEMENT

**Supplemental Table 1.** Patient-level and event level demographics.

|  | **Patient level** | **Event level** | |
| --- | --- | --- | --- |
|  | **n=15,800** | **n=18,194** | |
|  | **% (N)** | **% (N)** | |
| Female Gender | 38.4% (6,068) | Female Gender 38.6% (7,020) | |
| Black or African American race | 8.2% (1,302) | Black or African American race 8.6% (1,571) | |
| Hispanic ethnicity | 10.5% (1,660) | Hispanic ethnicity 10.5% (1,911) | |
| Age (at first heart attack) |  | Age (at this heart attack) | |
| <50 | 12.8% (2,026) | <50 | 12.5% (2,282) |
| 50-64 | 32.5% (5,131) | 50-64 | 32.3% (5,871) |
| 65-79 | 36.6% (5,776) | 65-79 | 36.9% (6,713) |
| 80+ | 18.2% (2,867) | 80+ | 18.3% (3,328) |
| Diabetes (at any heart attack) | 2.5% (393) | Diabetes (at this heart attack) | 1.9% (345) |
| HTN (at any heart attack) | 5.3% (844) | HTN (at this heart attack) | 3.8% (700) |
| Cigarettes (at any heart attack) | 29.4% (4,644) | Cigarettes (at this heart attack)  Highest creatinine > 1.5 | 29.2% (5,312)  29.7% (5,400) |
|  |  | Highest troponin |  |
|  |  | (0.04, 0.4] | 21.1% (3,833) |
|  |  | (0.4, 4] | 34.8% (6,326) |
|  |  | (4, 40] | 30.1% (5,485) |
|  |  | (40, 400] | 13.1% (2,382) |
|  |  | (400+) | 0.9% (168) |
